# FOR-HEALTH: study protocol for a randomized controlled feasibility trial of Structured Health Dialogues to improve lifestyle habits and metabolic health in forensic psychiatric inpatients

**DOI:** 10.1186/s40814-026-01919-2

**Published:** 2026-09-04

**Authors:** Mikael Vestlund, Veronica Milos Nymberg, Peter Andiné, Märta Wallinius

**Affiliations:** 1 Department of Clinical Sciences Lund, Psychiatry, Lund University, Lund, Sweden; 2 Research Department, Regional Forensic Psychiatric Clinic, Växjö, Sweden; 3 Center for Primary Health Care Research, Department of Clinical Sciences Malmö, Lund University, Malmö, Sweden; 4 University Clinic Primary Care, Skåne University Hospital, Region Skåne, Malmö, Sweden; 5 Centre for Ethics, Law and Mental Health, Department of Psychiatry and Neuochemistry, Institute of Neuroscience and Physiology, Sahlgrenska Academy, University of Gothenburg, Gothenburg, Sweden; 6 ReLife Centre, Lund University, Lund, Sweden; 7 Department of Forensic Psychiatry, Sahlgrenska University Hospital, Region Västra Götaland, Gothenburg, Sweden; 8 Department of Forensic Psychiatry, National Board of Forensic Medicine, Gothenburg, Sweden

**Keywords:** Forensic psychiatry, Severe mental illness, Cardiometabolic health, Lifestyle intervention, Physical health, Randomized controlled trial, Preventive care

## Abstract

**Background:**

People with severe mental illness demonstrate markedly reduced life expectancy, largely due to preventable cardiometabolic disease. This burden is particularly pronounced in compulsory forensic psychiatric care, where prolonged inpatient stays, restrictive environments, and long-term antipsychotic treatment contribute to high levels of sedentary behavior, unhealthy diet, tobacco use, and metabolic disturbances. Despite substantial cardiometabolic risk, individuals in forensic psychiatric settings are frequently excluded from preventive health research, resulting in limited evidence regarding the feasibility and acceptability of lifestyle interventions in this context. Recent international recommendations emphasize the need to integrate structured lifestyle interventions as core components of mental healthcare, including in secure and compulsory settings.

**Objectives:**

The FOR-HEALTH study aims to assess the feasibility and acceptability of an adapted Structured Health Dialogue intervention delivered in compulsory forensic psychiatric inpatient care. Secondary objectives are to describe cardiometabolic, lifestyle, and patient-reported outcomes to inform the design of a future definitive randomized controlled trial.

**Methods:**

This randomized controlled feasibility trial evaluates an adapted version of Structured Health Dialogues, an evidence-based cardiovascular prevention model originally developed for Swedish primary care. Participants are randomized in a 1:1 ratio to receive either Structured Health Dialogues plus treatment as usual or treatment as usual alone. Structured Health Dialogues integrate a systematic assessment of lifestyle behaviors, biological risk markers, psychosocial stress, and heredity into an individualized visual risk profile, which forms the basis for a motivational, person-centered dialogue. The intervention is adapted to the forensic psychiatric context and delivered within a secure inpatient setting. Primary outcomes relate to feasibility, including recruitment, retention, intervention delivery, and acceptability. Secondary outcomes include exploratory changes in lifestyle behaviors, cardiometabolic risk markers, and patient-reported outcomes. A total of 50 participants (25 per group) will be recruited. The study has been approved by the Swedish Ethical Review Authority. The findings are expected to inform the design and implementation of a future definitive randomized controlled trial evaluating Structured Health Dialogues in forensic psychiatric inpatient care.

**Trial registration:**

NCT07157813.

## Strengths and limitations of this study

### Strengths

#### High clinical relevance

The study addresses a well-documented physical health inequality among individuals with severe mental illness in compulsory forensic psychiatric care, a population that remains underrepresented in preventive health research.

#### Manualized, theory informed intervention

Structured Health Dialogue is a standardized, evidence-based intervention integrating cardiometabolic risk assessment, motivational interviewing, and visual risk communication, supporting reproducibility and future implementation.

#### Person-centered focus in a coercive context

Despite being conducted within compulsory forensic psychiatric care, the intervention explicitly emphasizes autonomy, shared decision-making, and individualized goal setting, addressing key ethical challenges in forensic psychiatric settings.

#### Pragmatic design

The trial is embedded in routine forensic psychiatric inpatient care, enhancing ecological validity and relevance for future scale up and real-world implementation.

#### Comprehensive outcome framework

Feasibility outcomes are combined with objective cardiometabolic measures and validated patient-reported outcomes, allowing assessment of both implementation processes and clinically relevant secondary outcomes.

## Limitations

### Single-center setting

Findings may have limited generalizability to other forensic psychiatric contexts with different organizational structures or patient populations.

### Feasibility-oriented primary aim

Although a power calculation was performed to enable detection of changes in selected secondary outcomes, the study is primarily designed to evaluate feasibility and acceptability. As such, findings related to clinical outcomes should be interpreted cautiously.

### Open-label design

Blinding of participants and clinicians is not feasible, which may introduce performance or expectancy bias.

### Contextual constraints

The secure inpatient environment may restrict participants’ ability to independently implement and sustain lifestyle changes beyond the intervention context.

### Limited long-term follow-up

Outcomes are assessed up to 12 months; longer-term sustainability of behavioral and metabolic changes cannot be determined.

## Introduction

### Background and rationale

People living with severe mental illness (SMI), including schizophrenia spectrum disorders, bipolar disorder, and major depressive disorder, have substantially reduced life expectancy and poorer physical health than the general population. The estimated mortality gap is approximately 15–20 years, with recent meta-analytic evidence confirming persistently increased mortality in schizophrenia and related disorders [1–4]. This excess mortality is largely driven by somatic diseases rather than external causes, with cardiovascular disease representing a major contributor to the mortality gap [5].

Cardiometabolic risk factors are highly prevalent among individuals with SMI. Metabolic syndrome, a cluster of metabolic abnormalities that increases the risk of both type 2 diabetes and cardiovascular disease, occurs substantially more frequently in people with SMI than in the general population [6, 7]. Diabetes mellitus is also more common among individuals with SMI [8] and data from the Swedish National Forensic Psychiatric Quality Register further illustrate the magnitude of this metabolic burden in forensic psychiatric populations, showing that diabetes is approximately 6.7 times more common among men and 9 times more common among women receiving forensic psychiatric care compared with the age-matched general population [9].

The excess physical health burden in SMI is widely recognized as multifactorial. Long-term exposure to psychotropic medication, particularly second-generation antipsychotics, is associated with weight gain and metabolic disturbances [10, 11]. Sedentary behavior is common among people with severe mental illness [12, 13], dietary patterns are often characterized by poor nutritional quality [14], and tobacco use remains highly prevalent [15]. Recent meta-reviews further support the efficacy and implementation of lifestyle and physical activity interventions as adjunctive treatments in severe mental illness [16].

Despite the high prevalence of modifiable risk factors, preventive somatic healthcare remains insufficiently integrated into routine psychiatric care. People with SMI are more likely to live with multiple coexisting physical health conditions (physical multimorbidity) [17] yet are less likely to receive systematic cardiovascular risk assessment and coordinated management of these conditions. Addressing physical health has therefore increasingly been recognized as an essential component of treatment and rehabilitation within psychiatric and forensic psychiatric services [18].

The Lancet Psychiatry Commission highlighted protection of physical health as a core component of mental healthcare [19]. More recently, the third report of the Lancet Psychiatry Physical Health Commission emphasized the need to implement structured interventions targeting physical activity, diet, and smoking within routine mental health services [20]. WHO recommendations similarly support integrating preventive physical healthcare into services for people with SMI [21].

Individuals receiving forensic psychiatric care typically present with complex SMI, most commonly schizophrenia spectrum disorders, often accompanied by substance use disorders and other psychiatric comorbidities [22]. These patients have committed criminal offenses in the context of SMI and receive compulsory treatment within secure psychiatric services where care is shaped by both therapeutic needs and considerations of risk and public safety. Registry data from Sweden further indicate substantial somatic health needs among individuals in forensic psychiatric care as well as long treatment trajectories within these services [23, 24]. Recent cohort and registry-based studies have also highlighted elevated mortality and adverse outcomes in forensic psychiatric populations [25, 26].

However, evidence regarding effective lifestyle interventions in forensic psychiatric populations remains limited. A systematic review concluded that although unhealthy lifestyle behaviors are common among forensic mental health service users, intervention studies targeting these behaviors in forensic settings remain scarce [27]. At the same time, initiatives aimed at improving somatic healthcare in forensic psychiatric settings are emerging. Recent work has explored the integration of primary care within specialized forensic psychiatric services [28], and intervention-oriented research is beginning to develop, including feasibility trials of structured weight management and lifestyle interventions [29, 30].

Structured Health Dialogues (SHD) represent a person-centered preventive approach developed for Swedish primary care. The method combines systematic assessment of lifestyle behaviors and biological risk markers with an individualized visual risk profile that supports dialogue and goal setting. Population-based evidence suggests that SHD may improve cardiovascular risk profiles and contribute to reduced mortality [31]. However, to our knowledge, SHD has not previously been evaluated in forensic psychiatric populations. Although SHD was originally developed and evaluated in Swedish primary care, implementation in compulsory forensic psychiatric inpatient care presents unique challenges. Compared with primary care, forensic psychiatric care is characterized by secure environments, restricted patient autonomy, and institutional routines that may influence both the delivery of the intervention and patients’ opportunities to modify lifestyle behaviors. Consequently, adaptation of both the intervention and the study procedures is required. A randomized controlled feasibility trial is therefore warranted to evaluate the feasibility and acceptability of the intervention and study procedures before conducting a future definitive randomized controlled trial. The overall aim of the FOR-HEALTH project is to improve the physical health of forensic psychiatric inpatients by developing and evaluating structured lifestyle interventions. The present randomized controlled feasibility trial aims to evaluate the feasibility and acceptability of implementing Structured Health Dialogues (SHD) in forensic psychiatric inpatient care and to generate data that will inform the design of a future definitive randomized controlled trial through exploratory evaluation of clinical outcomes.

## Aims and objectives

### Primary aim

To assess the feasibility and acceptability of SHD as a complement to treatment as usual (TAU) in forensic psychiatric inpatient services.

### Secondary aims

To describe cardiometabolic, lifestyle, and patient-reported outcomes, including potential changes in a composite cardiometabolic risk profile derived from SHD (the Swedish “health curve”), as well as metabolic risk markers.

## Methods and analysis

### Study design and setting

This study is a single-center, parallel-group, randomized controlled feasibility trial. The design and reporting follow methodological guidance for pilot and feasibility studies and adhere to the CONSORT 2010 statement and its extension for randomized pilot and feasibility trials [32, 33]. Participants are randomized in a 1:1 ratio to receive either SHD in addition to TAU or TAU alone. A total of 50 participants (25 per group) will be recruited.

**Fig. 1:**
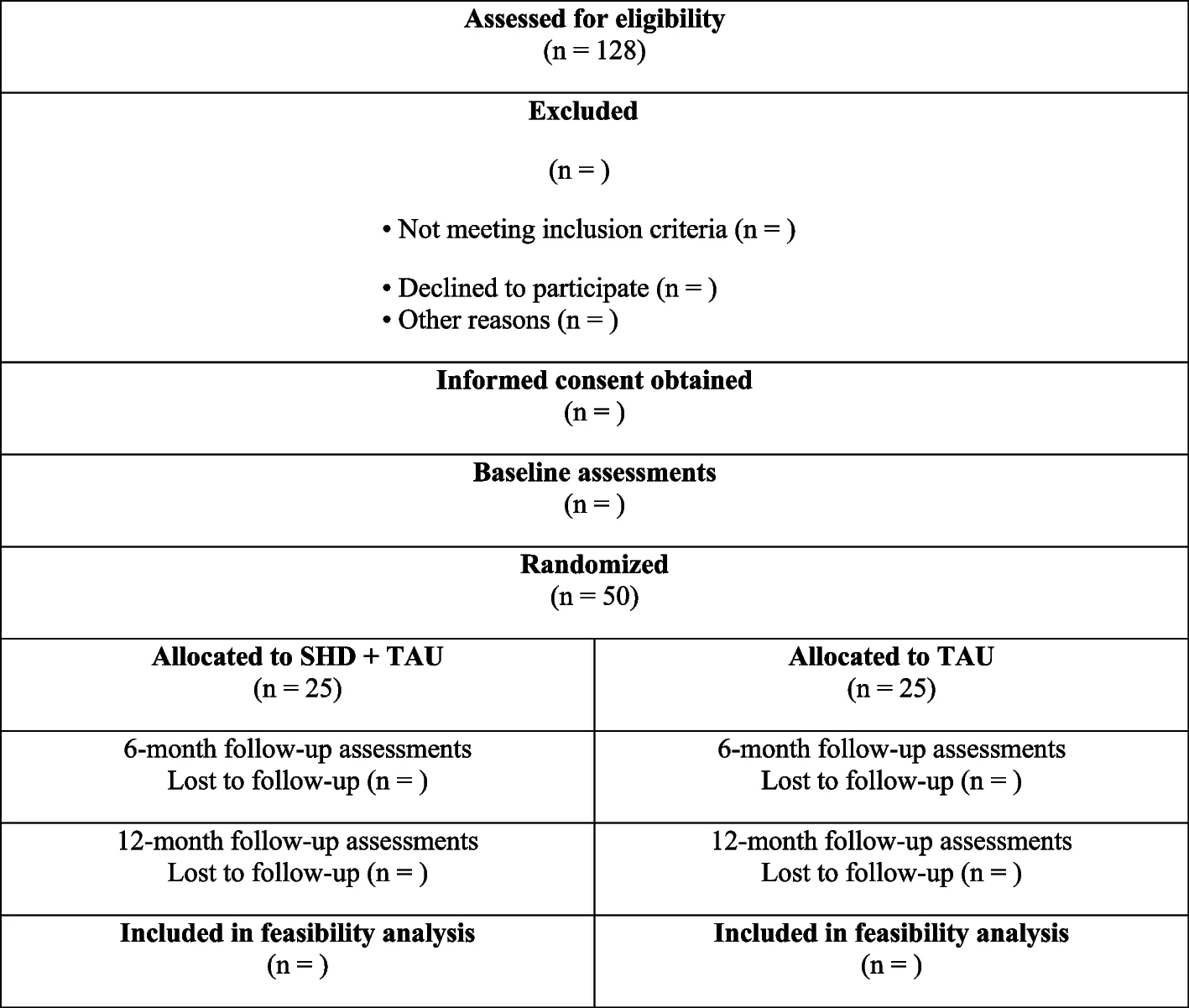
CONSORT flow diagram illustrating participant enrollment, randomization, follow-up, and feasibility analysis in the FOR-HEALTH trial

The overall flow of participants through the study, including enrollment, allocation, follow-up, and analysis, is illustrated in the CONSORT flow diagram (Fig. 1).

### Study setting

The trial is conducted at a large forensic psychiatric clinic in Sweden, providing compulsory inpatient care under the Swedish Forensic Psychiatric Care Act. Patients admitted to forensic psychiatric care have been convicted of criminal offenses and have been found to suffer from an SMI at the time of the offense. Therefore, they are sentenced to forensic psychiatric care rather than imprisonment. Care is individualized and may continue for several years depending on the patient’s psychiatric condition and the assessed risk of serious reoffending. The current clinic provides care across all three national security levels (levels 1–3), reflecting the full spectrum of Swedish forensic psychiatric inpatient care [24].

### Participants

#### Inclusion criteria

- Adult age, 18–64 years
- Admitted to forensic psychiatric inpatient care at the participating clinic
- Capacity to provide informed consent

#### Exclusion criteria

- Severe language barriers precluding meaningful participation
- Severe cognitive impairment precluding informed consent
- Acute psychiatric instability (e.g., acute psychosis or high suicide risk)
- Severe aggression or behavioral disturbance preventing safe participation

### Recruitment and consent

Potential participants are identified by clinical care teams and informed about the study. Written and verbal information is provided by the research team, and written informed consent is obtained prior to baseline assessments. Participation is voluntary and may be withdrawn at any time without consequences for ongoing care or legal status.

### Randomization and blinding

Randomization is performed after completion of baseline assessments using a computer-generated randomization sequence with block randomization, stratified by ward security level (security classes I–III). Stratification by ward security level was chosen because patients treated in different security levels may differ regarding psychiatric severity, level of supervision, access to rehabilitation activities, and opportunities to engage in lifestyle-related interventions, all of which may influence the feasibility and delivery of the intervention. Participants are not matched, as randomization is considered sufficient to ensure group comparability in this feasibility trial. An independent digital randomization table is generated using a secure web-based randomization service (www.sealedenvelope.com) to ensure allocation concealment prior to assignment.

The randomization sequence is generated in advance and implemented by a member of the research team not involved in recruitment or baseline assessment. Due to the nature of the intervention, the study is open label. Where feasible, data analysis will be conducted without knowledge of group allocation.

### Intervention

#### Structured Health Dialogues (SHD)

Participants allocated to the intervention group receive two nurse-led SHD sessions over a 12-month period. The first session is delivered at baseline following randomization, and the second session at 12-month follow-up. Participants in the intervention group also complete the scheduled follow-up assessments at 6 months, including anthropometric measurements, blood sampling, and patient-reported outcome measures. These assessments are performed to evaluate participant retention, completeness of data collection, and the feasibility of repeated outcome assessments, while also providing exploratory longitudinal outcome data. No additional SHD is delivered at the 6-month follow-up. Between study visits, participants continue to receive routine forensic psychiatric care according to local clinical practice, including standard psychiatric and medical care as clinically indicated. The SHD is intended to complement this ongoing care by providing individualized cardiovascular risk visualization and a structured person-centered dialogue to support patients’ engagement in lifestyle change. No additional SHD is delivered outside the study protocol.

Each session lasts approximately 60–90 min and includes:

- Visualization of individual cardiovascular and metabolic risk using the health curve, a graphical and pedagogical tool that integrates multiple lifestyle factors and biological risk markers into a color-coded risk profile (green to red), used to facilitate risk communication and support the person-centered health dialogue [34]
- A person-centered dialogue guided by motivational interviewing principles, focusing on autonomy, risk awareness, goal setting, and realistic behavioral change

### Control condition

Participants allocated to the control group will receive TAU provided at the forensic psychiatric clinic. TAU consists of multidisciplinary psychiatric care delivered according to individual clinical needs and may include pharmacological treatment, psychological support, occupational therapy, physiotherapy, social work, and other rehabilitation interventions. Health-related and lifestyle issues may be addressed as part of routine clinical care when considered clinically appropriate. However, participants in the control group will not receive SHD or the individualized cardiovascular risk visualization included in the intervention but will undergo the same assessments at baseline, 6 months, and 12 months as the intervention group.

### Assessments

Assessments are conducted at baseline, 6-month follow-up, and 12-month follow-up and include:

- Anthropometric measurements (body weight, height, BMI, waist-to-hip ratio) [35–37]
- Blood pressure measurement
- Blood sampling (fasting plasma glucose, HbA1c, lipid profile)
- A structured lifestyle questionnaire covering physical activity, diet, tobacco use, and alcohol consumption
- Health-related quality of life assessed using EQ-5D-5L [38–40]
- Mental fatigue assessed using the Mental Fatigue Scale (MFS) [41, 42]

These assessments are performed independently of the intervention sessions and are used to evaluate changes in cardiometabolic risk and lifestyle factors over time.

### Outcome measures

Outcome measures are derived from the assessments described above and are summarized in Table 1. A key exploratory outcome is change in the health curve, as described above. For analytical purposes, improvement in the health curve is defined as a shift of at least one risk category between baseline and follow-up.

**Table 1:** Study aims, objectives, and outcome measures in the FOR-HEALTH feasibility trial

Research question/aim(s)	Objectives	Outcomes
Primary	To assess the acceptability and feasibility of the research trial, including associated processes related to the intervention and assessments	Recruitment rates; follow-up retention and questionnaire/outcome response rates; adherence to Structured Health Dialogue sessions; completeness of data collection; assessment of safety (SAEs)
Secondary	Physical health: metabolic and cardiovascular risk factors Mental health: quality of life and mental fatigue Behavioral: lifestyle behaviors	BMI; blood pressure; waist–hip ratio; fasting glucose and HbA1c; lipid profile; EQ-5D-5L; Mental Fatigue Scale; physical activity, diet, tobacco and alcohol use
Tertiary	To inform the design of a future definitive randomized controlled trial	Feasibility parameters; variability and preliminary estimates of outcomes; data completeness and acceptability of outcome measures

*BMI* body mass index, *HbA1c* glycated hemoglobin, *EQ-5D-5L* EuroQol 5 Dimensions 5 Levels, *SAEs* serious adverse events

### Data collection and assessment schedule

**Fig. 2:**
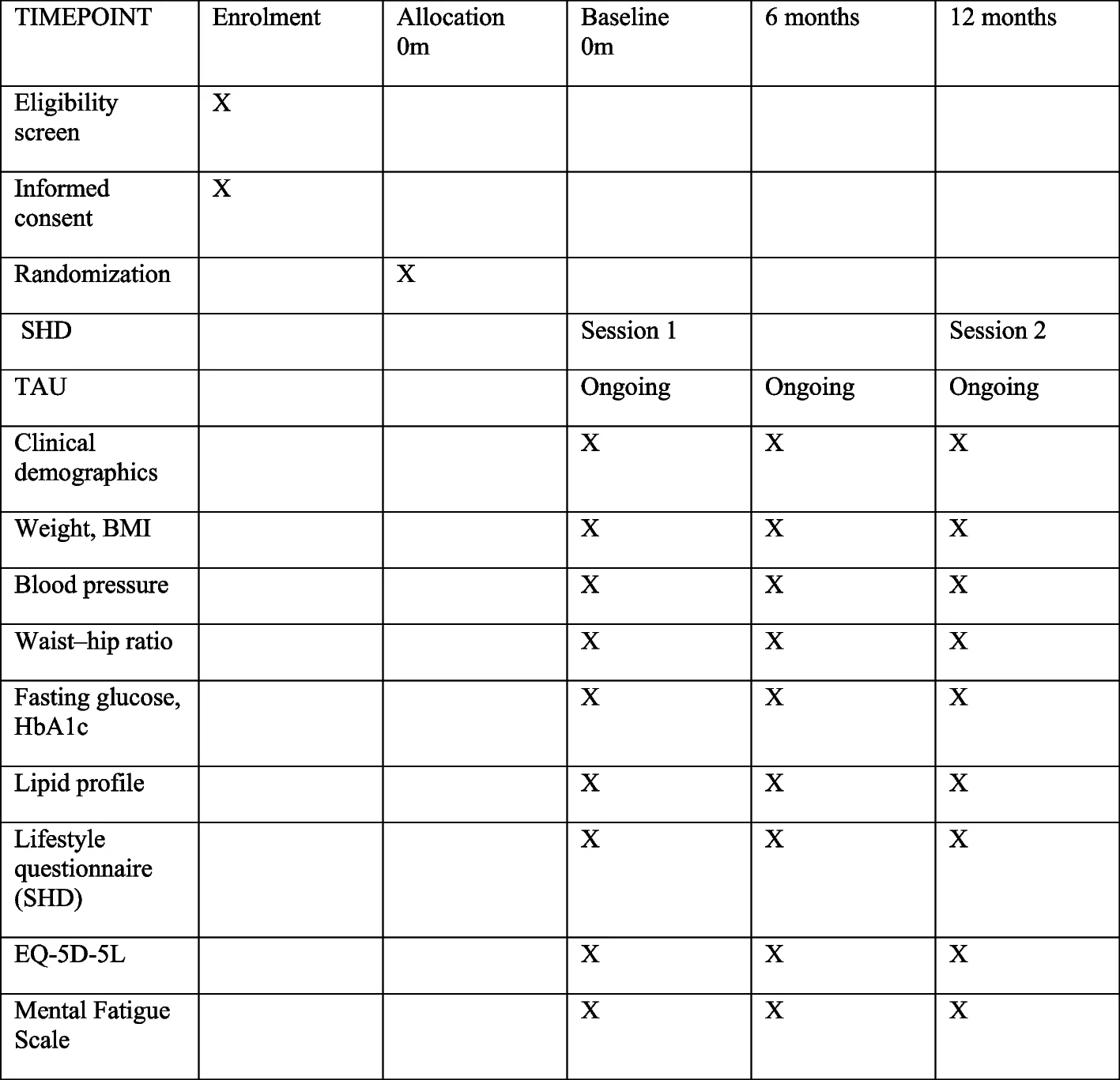
SPIRIT schedule of enrollment, intervention delivery, and outcome assessments in the FOR-HEALTH feasibility trial. SHD Structured Health Dialogue, TAU treatment as usual, BMI body mass index, HbA1c glycated hemoglobin

The timing of enrollment, intervention delivery, and outcome assessments is illustrated in the SPIRIT schedule (Fig. 2).

### Sample size and statistical analysis

#### Sample size rationale

This study is designed as a randomized controlled feasibility trial with primary objectives focused on recruitment, retention, acceptability, intervention delivery, and completeness of data collection in compulsory forensic psychiatric care and a secondary, explorative evaluation of intervention impact on clinical outcomes. A total of 50 participants (25 per group) will be recruited.

A formal sample size calculation was undertaken based on a clinically meaningful change in the health curve, in order to evaluate a potential impact of the intervention on clinical outcomes related to the secondary, exploratory aim. Consistent with prior evaluations of the SHD model, a clinically relevant response was defined as a priori as an improvement of at least one risk category between baseline and follow-up. The proportion of participants achieving such an improvement was conservatively assumed to be 0% in the control group and 30% in the intervention group. Using a two-sided significance level of 5% (*α* = 0.05) and a power of 90% (1 − *β* = 0.90), this corresponds to a required sample size of 25 participants per group.

Although this calculation indicates that the planned sample allows preliminary exploration of change in this outcome, the study is not intended to provide conclusive evidence of effectiveness. Analyses of clinical outcomes will therefore be interpreted as exploratory and used to inform the design of a future definitive randomized controlled trial.

#### Statistical analysis

Analyses will be conducted using Stata (StataCorp, College Station, TX, USA) and will follow the intention-to-treat principle.

Feasibility outcomes, including recruitment, retention, intervention adherence, and data completeness, will be summarized using descriptive statistics with corresponding 95% confidence intervals.

Continuous outcomes will be analyzed using linear mixed-effects models, including fixed effects for group, time, and group-by-time interaction, and random effects to account for within-subject correlation over time. Where appropriate, model assumptions will be assessed and alternative analytical approaches considered if necessary.

Results will be presented as estimated effect sizes with 95% confidence intervals. Given the feasibility nature of the study, no formal hypothesis testing is planned, and *p *values will be interpreted with caution.

Given the study design, analyses of secondary outcomes are exploratory and are not intended to evaluate intervention effectiveness. Instead, they are intended to estimate outcome variability and to inform the design, outcome selection, and sample size calculation of a future definitive randomized controlled trial. The extent, pattern, and completeness of outcome data will be described for each outcome and follow-up assessment and will be considered when interpreting the feasibility of the study procedures.

### Patient and public involvement

The study is informed by extensive clinical experience in forensic psychiatric care, which has guided the adaptation of the intervention and study procedures to the specific organizational and clinical conditions of compulsory forensic psychiatric inpatient care.

Patient and public involvement has been incorporated during the study planning phase and will be continuously applied through collaboration ReLife Centre at Lund University.

Findings from the study will be fed back to clinical services and are intended to support the development and implementation of sustainable preventive health interventions within forensic psychiatric settings.

### Ethics and dissemination

The study has been approved by the Swedish Ethical Review Authority. Particular attention is paid to voluntariness, autonomy, and protection of participant integrity in the compulsory care context. Results will be disseminated through peer-reviewed publications, conference presentations, and feedback on participating in clinical services.

## Trial status

Recruitment is planned to begin in February 2026 and will be conducted on a rolling basis within the participating forensic psychiatric inpatient clinic, with completion expected by December 2027. Follow-up assessments are conducted 6 and 12 months after baseline. Outcome data are expected to be collected by December 2028, with data analysis completed by the end of 2029.

## Data Availability

No datasets were generated or analysed during the current study.
